# Receptor-Targeted Peptide Conjugates Based on Diphosphines Enable Preparation of ^99m^Tc and ^188^Re Theranostic Agents for Prostate Cancer

**DOI:** 10.2967/jnumed.124.267450

**Published:** 2024-07

**Authors:** Truc T. Pham, Ingebjørg N. Hungnes, Charlotte Rivas, Julie Cleaver, George Firth, Philip J. Blower, Jane Sosabowski, Gary J.R. Cook, Lefteris Livieratos, Jennifer D. Young, Paul G. Pringle, Michelle T. Ma

**Affiliations:** 1 School of Bioengineering and Imaging Sciences, St. Thomas’ Hospital, King’s College London, London, United Kingdom;; 2 Centre for Cancer Biomarkers and Biotherapeutics, Barts Cancer Institute, John Vane Science Centre, Queen Mary University of London, London, United Kingdom;; 3 Department of Nuclear Medicine, Guy’s and St. Thomas’ Hospitals NHS Foundation Trust, Guy’s Hospital, London, United Kingdom; and; 4 School of Chemistry, University of Bristol, Bristol, United Kingdom

**Keywords:** ^99m^Tc, ^188^Re, SPECT, phosphine, PSMA

## Abstract

Benchtop ^99^Mo/^99m^Tc and ^188^W/^188^Re generators enable economical production of molecular theranostic ^99m^Tc and ^188^Re radiopharmaceuticals, provided that simple, kit-based chemistry exists to radiolabel targeting vectors with these radionuclides. We have previously described a diphosphine platform that efficiently incorporates ^99m^Tc into receptor-targeted peptides. Here, we report its application to label a prostate-specific membrane antigen (PSMA)–targeted peptide with ^99m^Tc and ^188^Re for diagnostic imaging and systemic radiotherapy of prostate cancer. **Methods:** Two diphosphine-dipeptide bioconjugates, DP1-PSMAt and DP2-PSMAt, were formulated into kits for radiolabeling with ^99m^Tc and ^188^Re. The resulting radiotracers were studied in vitro, in prostate cancer cells, and in vivo in mouse xenograft models, to assess similarity of uptake and biodistribution for each ^99m^Tc/^188^Re pair of agents. **Results:** Both DP1-PSMAt and DP2-PSMAt could be efficiently radiolabeled with ^99m^Tc and ^188^Re using kit-based methods to furnish the isostructural compounds M-DP1-PSMAt and M-DP2-PSMAt (M = [^99m^Tc]Tc, [^188^Re]Re). All ^99m^Tc/^188^Re radiotracers demonstrated specific uptake in PSMA-expressing prostate cancer cells, with negligible uptake in prostate cancer cells that did not express PSMA or in which PSMA uptake was blocked. M-DP1-PSMAt and M-DP2-PSMAt also exhibited high tumor uptake (18–30 percentage injected dose per gram at 2 h after injection), low retention in nontarget organs, fast blood clearance, and excretion predominantly via a renal pathway. Importantly, each pair of ^99m^Tc/^188^Re radiotracers showed near-identical biologic behavior in these experiments. **Conclusion:** We have prepared and developed novel pairs of isostructural PSMA-targeting ^99m^Tc/^188^Re theranostic agents. These generator-based theranostic agents have potential to provide access to the benefits of PSMA-targeted diagnostic imaging and systemic radiotherapy in health care settings that do not routinely have access to either reactor-produced ^177^Lu radiopharmaceuticals or PET/CT infrastructure.

## Visual Abstract

**Figure:**
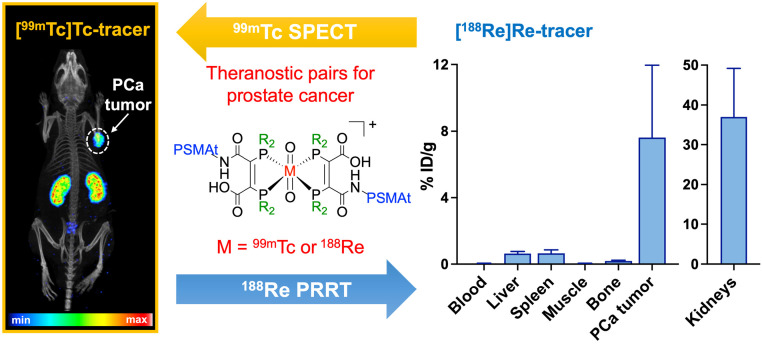

The PSMAt peptide, which targets the prostate-specific membrane antigen (PSMA), has had clinical impact as a vector for delivering radionuclides to prostate cancer for diagnostic imaging and systemic peptide receptor radionuclide therapy (PRRT). The radiopharmaceutical [^177^Lu]Lu-PSMA-617 (1,2) has recently been approved by the Food and Drug Administration for PRRT of metastatic castration-resistant prostate cancer. Diagnostic PET imaging with [^68^Ga]Ga-PSMA-11 can inform clinical decision-making for treatment of prostate cancer, and [^68^Ga]Ga-PSMA-11 is widely used as a diagnostic companion to [^177^Lu]Lu-PSMA-617 (3). PSMA-targeted radiopharmaceuticals for SPECT/γ-scintigraphy imaging, such as [^99m^Tc]Tc-MIP-1404 (4,5), [^99m^Tc]Tc-PSMA-I&S (6,7), and [^99m^Tc]Tc-EDDA/HYNIC-iPSMA (8,9), have been developed as alternatives to ^68^Ga to enhance access to PSMA scanning when ^68^Ga and PET are less accessible but generator-based ^99m^Tc and SPECT/γ-scintigraphy cameras are available. Although these ^99m^Tc (half-life of 6 h, 90% γ, 140 keV) radiotracers exhibit lower sensitivity than PSMA-targeted PET radiotracer alternatives, particularly in the case of biochemical recurrence of prostate cancer at low PSA levels or low tumor volumes, they have potential utility in providing useful diagnostic information at high tumor volumes, assessing suitability for and response to PRRT and radioguided surgery for which detection of every site of small volume of disease is less critical (4,7,8).

A range of radiometal ions with therapeutically efficacious emission profiles (e.g.,^225^Ac, ^227^Th, ^212^Pb, and ^161^Tb) has been used as alternatives to ^177^Lu. However, in lower- and middle-income countries (LMICs), the availability of PRRT is limited by cost. The batch-produced radiopharmaceuticals that prevail in high-income countries are prohibitively expensive and are available only to the wealthiest patients in LMICs. Additionally, the availability of PET infrastructure, including cyclotrons and scanners, in LMICs is limited (10).

The chemistry of rhenium is closely similar to that of its lighter congener technetium. Importantly, β^−^-emitting ^188^Re (half-life of 17 h, 100% β^−^, 2.12 MeV, 15% γ, 155 keV) is available from a benchtop ^188^W/^188^Re generator, offering hospitals an economical and routinely accessible source of a therapeutic radionuclide. Radiopharmaceuticals based on the theranostic ^99m^Tc/^188^Re pair offer a potentially viable solution to economic and geographic barriers posed by existing theranostic PRRT (11–13). Traditional ^99m^Tc-radiopharmaceuticals are used in 30 million scans worldwide per year, including in LMICs, in combination with γ-scintigraphy (10,14). Additionally, international consortia have identified ^188^Re as a highly promising basis for systemic radiotherapy in LMICs: recognizing the affordability of ^188^Re, the International Atomic Energy Agency has sponsored multinational clinical trials of ^188^Re-labeled Lipiodol for treatment of inoperable liver cancer in LMICs (11). ^188^Re-labeled Lipiodol proved effective and inexpensive. ^188^Re-labeled bisphosphonates have also been extremely beneficial in palliative treatment of bone metastases in LMICs (15). There is high potential for the combination of ^99m^Tc with ^188^Re to provide economical, population-wide access to stratified molecular imaging and PRRT in LMICs, provided that suitable chemical platforms are available to enable radiochemical production of well-defined pairs of theranostic ^99m^Tc/^188^Re agents.

A new theranostic pair of ^99m^Tc/^188^Re-labeled peptide radiotracers has recently demonstrated preclinical and clinical PSMA-targeting efficacy in prostate cancer (16), highlighting the potential utility of this theranostic approach. We have contemporaneously developed 2 kit-based ^99m^Tc-radiolabeled agents, [^99m^Tc]Tc-DP1-PSMAt and [^99m^Tc]Tc-DP2-PSMAt (Fig. 1; Supplemental Chart 1; supplemental materials are available at http://jnm.snmjournals.org.), targeting PSMA and reported their chemical properties (17). These compounds are based on a diphosphine chelator (18), which also coordinates Re^V^: we have used analogous nonradioactive Re^V^ derivatives to chemically characterize the radioactive Tc^V^ compounds (17–19). Here, we report the preparation of isostructural and isoelectronic ^188^Re-labeled derivatives ([^188^Re]Re-DP1-PSMAt and [^188^Re]Re-DP2-PSMAt), the biologic behavior of the novel ^99m^Tc radiotracers in prostate cancer models, and the comparative biologic behavior of ^188^Re compounds.

**FIGURE 1.**
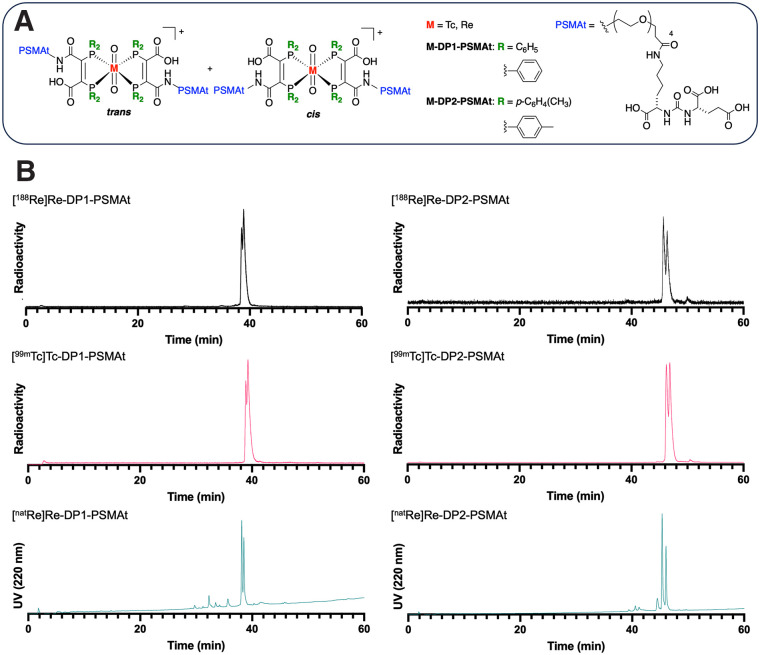
(A) Chemical structures of M-DP1-PSMAt and M-DP2-PSMAt, M = Tc (^99m^Tc or ^99g^Tc) and Re (^nat^Re or ^188^Re). (B) Reverse-phase C18-HPLC radiochromatograms showing coelution of [^188^Re]Re-DP1-PSMAt, [^99m^Tc]Tc-DP1-PSMAt, and [^nat^Re]Re-DP1-PSMAt and coelution of [^188^Re]Re-DP2-PSMAt, [^99m^Tc]Tc-DP2-PSMAt, and [^nat^Re]Re-DP2-PSMAt. UV = ultraviolet.

## MATERIALS AND METHODS

### ^188^Re Radiolabeling

[^188^Re]Re^V^-citrate was prepared from a saline solution of preconcentrated (13) [^188^Re]ReO_4_^−^ in more than 95% yield (20,21). An aliquot of this solution (75 μL) was added to a DP1-PSMAt kit or DP2-PSMAt kit (Supplemental Table 1), which was heated at 90°C for 30 min. The reaction solution was then analyzed by C18 radio–high-performance liquid chromatography (HPLC). [^188^Re]Re-DP1-PSMAt eluted at 12.7 min, and [^188^Re]Re-DP2-PSMAt eluted at 17.5 min (Supplemental Fig. 1). The reaction mixtures, containing either [^188^Re]Re-DP1-PSMAt or [^188^Re]Re-DP2-PSMAt, were purified and reformulated for biologic experiments.

### ^99m^Tc and ^188^Re Radiotracer Uptake in Prostate Cancer Cells

Solutions containing radiotracer (100 kBq in 8–12 μL of phosphate-buffered saline, >95% radiochemical purity) were added to DU145-PSMA+ or LNCaP prostate cancer cells (22), and the cells were incubated at 37°C for 1 h. Nonspecific uptake was determined using non–PSMA-expressing cells (DU145, PC3) or by blocking PSMA-expressing cells (DU145-PSMA+, LNCaP) with 2-phosphonomethyl pentanedioic acid (PMPA). After incubation, the cells were washed and samples collected for radioactivity counting. Uptake and localization of ^99m^Tc radiotracers were measured over time (15, 30, 60, and 120 min) in DU145-PSMA+ or LNCaP cells (supplemental materials).

### SPECT/CT Scanning and Biodistribution Studies in Mice

All animal experiments were ethically reviewed by an Animal Welfare and Ethical Review Board at either King’s College London or Barts Cancer Institute and were performed in accordance with the Animals (Scientific Procedures) Act 1986 U.K. Home Office regulations governing animal experimentation. Subcutaneous prostate cancer DU145-PSMA+ or DU145 xenografts were produced in SCID/beige mice (male, 7–12 wk old). Subcutaneous LNCaP prostate cancer xenografts were produced in athymic nude mice (Crl:NU(NCr)-*Foxn1^nu^*, male, 6–7 wk old). SPECT/CT and biodistribution studies using ^99m^Tc and ^188^Re radiotracers were performed once a tumor had reached an appropriate size (supplemental materials).

## RESULTS

### Preparation of [^188^Re]Re-DP1-PSMAt and [^188^Re]Re-DP2-PSMAt

We have previously prepared [^99m^Tc]Tc-DP1-PSMAt and [^99m^Tc]Tc-DP2-PSMAt using a single-step radiolabeling kit (17). Here, the analogous ^188^Re agents were prepared in 2 steps from a saline solution containing [^188^Re]ReO_4_^−^, obtained from a ^188^W/^188^Re OncoBeta generator. In the first step, [^188^Re]ReO_4_^−^ was reduced to a [^188^Re]Re^V^-citrate precursor: an aqueous solution containing [^188^Re]ReO_4_^−^ (∼300–500 MBq), sodium citrate, and stannous chloride (SnCl_2_), at pH 5.5, was heated at 90°C for 30 min, to give [^188^Re]Re^V^-citrate in more than 95% yield, as previously described (20,21). After this, [^188^Re]Re^V^-citrate (∼130–210 MBq) was added to a prefabricated, lyophilized kit (Supplemental Table 1) containing sodium carbonate, sodium tartrate, SnCl_2_, and either DP1-PSMAt or DP2-PSMAt. The resulting radiolabeling solution (pH 8.0–8.5) was heated at 90°C for 30 min to form [^188^Re]Re-DP1-PSMAt in 20%–70% radiochemical yield and [^188^Re]Re-DP2-PSMAt in 20%–50% radiochemical yield (as determined by radio-HPLC): whereas formation of the desired products was reproducible, yields were not.

[^188^Re]Re-DP1-PSMAt and [^188^Re]Re-DP2-PSMAt were subsequently isolated using reverse-phase HPLC, lyophilized, and then reconstituted in phosphate-buffered saline to yield the radiotracers in more than 95% radiochemical purity. The purified ^188^Re compounds were analyzed by reverse-phase radio-HPLC. Each ^188^Re compound coeluted with its nonradioactive ^nat^Re isotopolog and radioactive ^99m^Tc analog (17), confirming not only the chemical identity of these new ^188^Re agents but also that they are isostructural and isoelectronic with their ^99m^Tc analogs (Fig. 1). Each rhenium and technetium compound consists of 2 closely eluting isomers, *cis-* and *trans*-[M^V^O_2_(DPX-PSMAt)_2_]^+^ (M = Re or Tc, X = 1 or 2) (17). The *cis* and *trans* designations denote the relative positions of the PSMAt moieties.

Solutions of each new ^188^Re radiotracer were added to human serum and incubated at 37°C for 24 h followed by analysis by reverse-phase analytic radio-HPLC, which demonstrated that [^188^Re]Re-DP1-PSMAt and [^188^Re]Re-DP2-PSMAt are stable, with more than 95% each radiotracer, respectively, observed intact in human serum over this time frame (Supplemental Fig. 2).

### Uptake of ^99m^Tc and ^188^Re Agents in Prostate Cancer Cells

**FIGURE 2.**
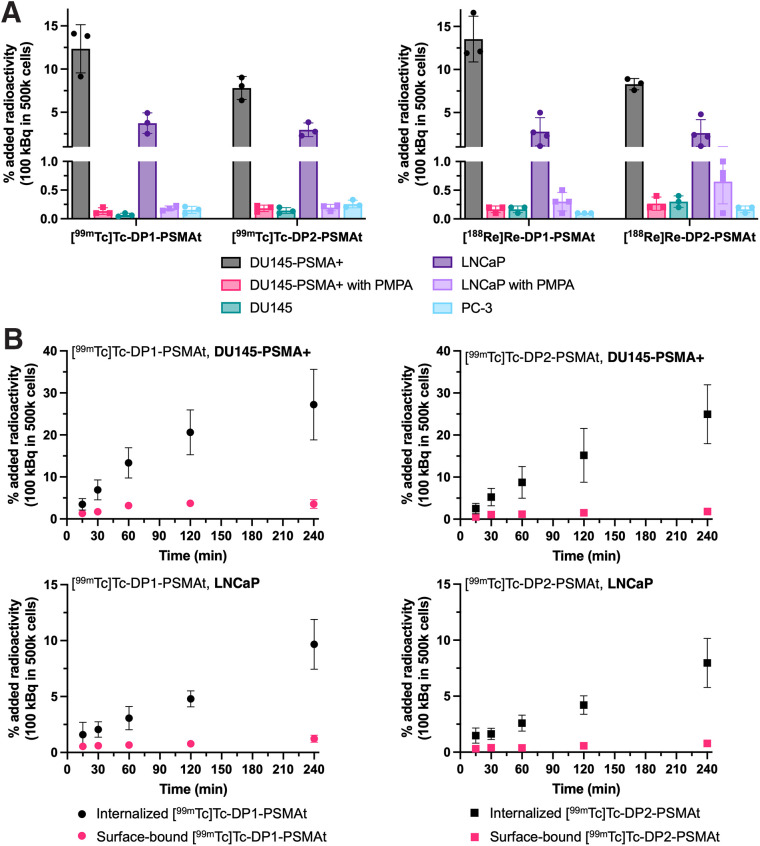
(A) Uptake of radiotracers in PSMA-positive and PSMA-negative prostate cancer cells. (B) Time course uptake and localization of ^99m^Tc radiotracers in DU145-PSMA cells and LNCaP cells. Data are presented as mean ± SD; *n* = 3–4 biologic repeats performed in triplicate.

To assess the specificity of the new radiotracers for PSMA, [^99m^Tc]Tc-DP1-PSMAt and [^99m^Tc]Tc-DP2-PSMAt (100 kBq) were each incubated with DU145-PSMA+ prostate cancer cells (DU145 cells transfected to express PSMA receptor) (22). After 1 h of incubation, uptake of each radiotracer was quantified. To assess specificity, each radiotracer was also coincubated with the PSMA inhibitor PMPA with DU145-PSMA+ cells and incubated with parental DU145 cells that do not express PSMA. [^99m^Tc]Tc-DP1-PSMAt and [^99m^Tc]Tc-DP2-PSMAt exhibited uptake in DU145-PSMA+ cells (12.4 ± 2.8 percentage added radioactivity [%AR] and 7.8 ± 1.3 %AR, respectively). This uptake was specific: DU145-PSMA+ cell uptake of [^99m^Tc]Tc-DP1-PSMAt and [^99m^Tc]Tc-DP2-PSMAt could be blocked with PMPA, and there was negligible uptake in parental DU145 cells (Fig. 2). The uptake of [^99m^Tc]Tc-DP1-PSMAt and [^99m^Tc]Tc-DP2-PSMAt was also studied in LNCaP prostate cancer cells, which natively express PSMA, and PC3 cells, which, like parental DU145 cells, do not express PSMA. In LNCaP cells, uptake of [^99m^Tc]Tc-DP1-PSMAt and [^99m^Tc]Tc-DP2-PSMAt measured 3.7 ± 1.2 %AR and 3.0 ± 0.8 %AR, respectively, whereas uptake of both radiotracers in PC3 cells measured less than 0.3 %AR. Uptake in LNCaP cells could also be blocked with PMPA.

The cellular localization of [^99m^Tc]Tc-DP1-PSMAt and [^99m^Tc]Tc-DP2-PSMAt was also evaluated in DU145-PSMA+ and LNCaP cells over time (Fig. 2). Uptake of both radiotracers increased over 2 h, and most cell-associated radioactivity was found in the internalized cell fraction at all measured time points for both PSMA-expressing cell lines. Uptake of [^99m^Tc]Tc-DP1-PSMAt (both surface-bound and internalized radioactivity) was slightly higher than that of [^99m^Tc]Tc-DP2-PSMAt.

Additionally, the uptake of [^188^Re]Re-DP1-PSMAt and [^188^Re]Re-DP2-PSMAt (100 kBq) was also assessed in the abovementioned prostate cancer cell lines: both radiotracers exhibited uptake in DU145-PSMA+ cells (13.5 ± 2.7 %AR and 8.3 ±0.7 %AR, respectively) and LNCaP cells (2.8 ± 1.6 %AR and 2.6 ± 1.6 %AR, respectively). This uptake was also specific: uptake of [^188^Re]Re-DP1-PSMAt and [^188^Re]Re-DP2-PSMAt could be blocked with PMPA, and there was negligible uptake in parental DU145 cells and PC3 cells (Fig. 2).

### Biodistribution and SPECT/CT Imaging of ^99m^Tc Radiotracers in Mice Bearing Prostate Cancer Tumors

**FIGURE 3.**
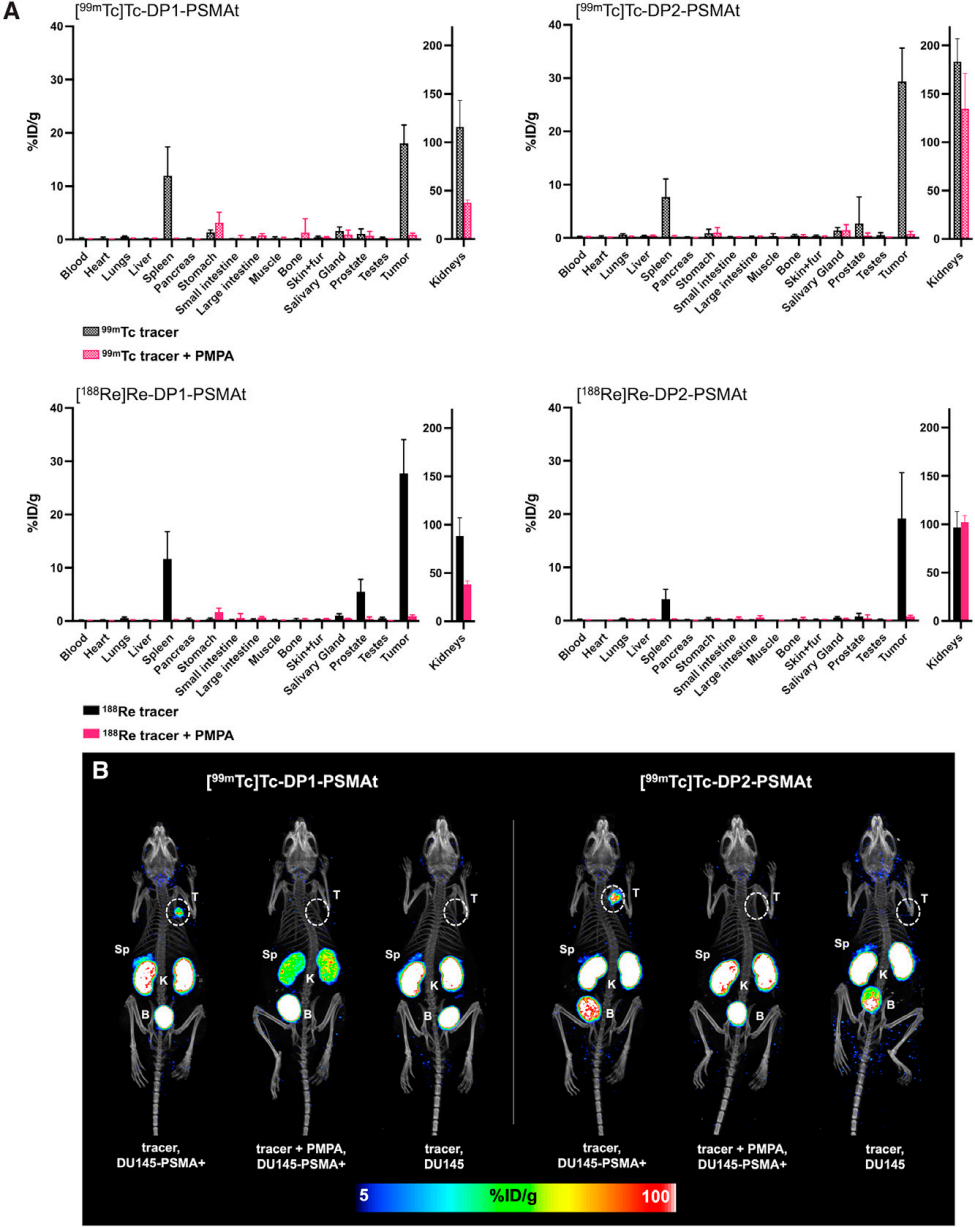
(A) Biodistribution (2 h after injection) of SCID/beige mice bearing DU145-PSMA+ prostate cancer tumors, administered either ^99m^Tc or ^188^Re radiotracers intravenously. To assess specificity of radiotracer uptake, additional groups of mice were also coadministered radiotracer and PMPA (Supplemental Tables 3 and 4). (B) Whole-body SPECT/CT maximum-intensity projections of SCID/beige mice bearing either DU145-PSMA+ tumors or DU145 tumors, administered either [^99m^Tc]Tc-DP1-PSMAt or [^99m^Tc]Tc-DP2-PSMAt, 2 h after injection. To inhibit uptake in DU145-PSMA+ tumors, animals were also coadministered PMPA.

The biodistributions of [^99m^Tc]Tc-DP1-PSMAt and [^99m^Tc]Tc-DP2-PSMAt were assessed in male SCID/beige mice bearing DU145-PSMA+ xenograft tumors (Fig. 3). Each mouse was administered either [^99m^Tc]Tc-DP1-PSMAt or [^99m^Tc]Tc-DP2-PSMAt and euthanized at 2 h after injection (*n* = 5), followed by organ harvesting for ex vivo radioactivity counting. High amounts of each tracer were observed in tumors 2 h after injection; for [^99m^Tc]Tc-DP1-PSMAt, the [^99m^Tc]Tc concentration measured 18.0 ± 3.5 percentage injected dose per gram (%ID/g), and for [^99m^Tc]Tc-DP2-PSMAt, the [^99m^Tc]Tc concentration measured 29.4 ± 6.3 %ID/g.

To assess the specificity of each radiotracer, separate groups of animals, also bearing DU145-PSMA+ tumors, were coadministered either [^99m^Tc]Tc-DP1-PSMAt and PMPA or [^99m^Tc]Tc-DP2-PSMAt and PMPA to inhibit PSMA-mediated uptake of radiotracer (*n* = 5). In mice bearing DU145-PSMA+ tumors, coadministration of PMPA substantially decreased uptake of both [^99m^Tc]Tc-DP1-PSMAt and [^99m^Tc]Tc-DP2-PSMAt in tumors. For [^99m^Tc]Tc-DP1-PSMAt, coadministration decreased uptake to 0.91 ± 0.29 %ID/g in tumors (compared with administration of [^99m^Tc]Tc-DP1-PSMAt only: mean difference of 17.12 %ID/g, *P* = 4 × 10^−4^). For [^99m^Tc]Tc-DP2-PSMAt, coadministration decreased uptake to 0.76 ± 0.45 %ID/g in tumors (compared with administration of [^99m^Tc]Tc-DP2-PSMAt only: mean difference of 28.62 %ID/g, *P* = 5 × 10^−4^).

For both radiotracers, the concentration of ^99m^Tc radioactivity in the kidneys 2 h after injection was high (Fig. 3). Increased amounts of [^99m^Tc]Tc-DP2-PSMAt were measured in the kidneys 2 h after injection (183.3 ± 23.8 %ID/g,) compared with [^99m^Tc]Tc-DP1-PSMAt (115.9 ± 27.3 %ID/g, mean difference of 67.4 %ID/g, *P* = 0.003). Notably, for animals administered [^99m^Tc]Tc-DP1-PSMAt, coadministration of PMPA significantly decreased retention of ^99m^Tc radioactivity in the kidneys. In contrast, although coadministration of PMPA also decreased the radioactivity concentration in the kidneys for animals injected with [^99m^Tc]Tc-DP2-PSMAt, this effect was much less pronounced. There were also significant amounts of both radiotracers ([^99m^Tc]Tc-DP1-PSMAt: 12.0 ± 5.3 %ID/g; [^99m^Tc]Tc-DP2-PSMAt: 7.7 ± 3.4 %ID/g) that residualized in the spleen, which is known to express low levels of PSMA and accumulate PSMA-targeted radiotracers (5,6,9). As expected, coadministration of PMPA significantly decreased retention of ^99m^Tc radioactivity in the spleen for both radiotracers ([^99m^Tc]Tc-DP1-PSMAt: 0.2 ± 0.06 %ID/g, mean difference of 11.8 %ID/g, *P =* 0.008; [^99m^Tc]Tc-DP2-PSMAt: 0.3 ± 0.18 %ID/g, mean difference of 7.4 %ID/g, *P =* 0.008).

Additionally, groups of mice bearing non–PSMA-expressing parental DU145 tumors were also administered these ^99m^Tc radiotracers. For these groups, tumor uptake of [^99m^Tc]Tc-DP1-PSMAt decreased to 0.24 ± 0.07 %ID/g, and tumor uptake of [^99m^Tc]Tc-DP2-PSMAt decreased to 0.18 ± 0.07 %ID/g (Supplemental Fig. 3).

In SPECT/CT scans of animals administered either [^99m^Tc]Tc-DP1-PSMAt or [^99m^Tc]Tc-DP2-PSMAt only, tumors could be clearly delineated at both 2 h (Fig. 3) and 24 h (Supplemental Fig. 4) after injection. The kidneys and bladder were also clearly visible across these time points, consistent with ex vivo biodistribution data. SPECT/CT also showed negligible tumor uptake for animals either coadministered PMPA or bearing DU145 tumors that do not express PSMA receptor (Fig. 3). For all animals administered either [^99m^Tc]Tc-DP1-PSMAt only or [^99m^Tc]Tc-DP2-PSMAt only, the spleen was also identified in SPECT/CT scans acquired at 2 h after injection. Coadministration of PMPA decreased spleen uptake of both radiotracers.

### Biodistribution of ^188^Re Radiotracers in Mice Bearing Prostate Cancer Tumors

The biodistributions of the ^188^Re radiotracers, [^188^Re]Re-DP1-PSMAt and [^188^Re]Re-DP2-PSMAt, were first assessed in SCID/beige mice bearing DU145-PSMA+ tumors (Fig. 3). Radioactivity concentration in the tumors of animals administered [^188^Re]Re-DP1-PSMAt measured 27.7 ± 6.4 %ID/g at 2 h after injection, whereas that of animals given [^188^Re]Re-DP2-PSMAt measured 19.2 ± 8.6 %ID/g. Both compounds cleared the circulation via a renal pathway, as evidenced by high concentrations of radioactivity measured in the kidneys ([^188^Re]Re-DP1-PSMAt measured 88.3 ± 18.8 %ID/g, [^188^Re]Re-DP2-PSMAt measured 96.8 ± 16.3 %ID/g). Biodistribution data also indicated that both compounds had low retention in nontarget, healthy organs and tissues, except for the spleen. In this experiment, there were no notable significant differences between the biodistribution profiles of [^188^Re]Re-DP1-PSMAt and [^188^Re]Re-DP2-PSMAt at 2 h after injection. As expected, coadministration of PMPA significantly inhibited uptake of both ^188^Re radiotracers in the tumor and spleen.

Urine was collected from mice administered either [^188^Re]Re-DP1-PSMAt or [^188^Re]Re-DP2-PSMAt at 2 h after injection and was analyzed by reverse-phase radio-HPLC. Radiochromatograms showed that both [^188^Re]Re-DP1-PSMAt and [^188^Re]Re-DP2-PSMAt were highly stable and were cleared from the blood pool and excreted chemically intact (Supplemental Fig. 5).

**FIGURE 4.**
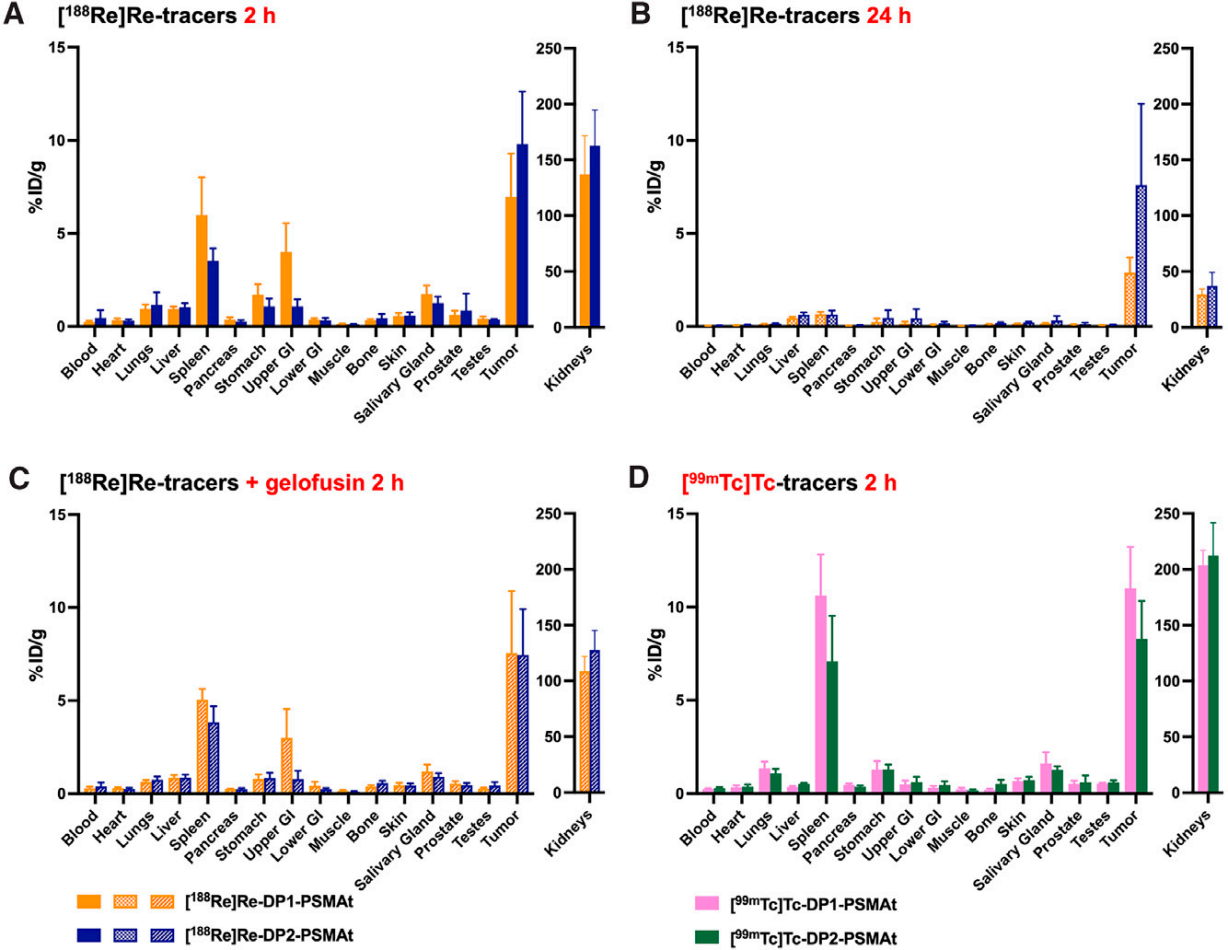
(A–C) Biodistribution of ^188^Re in athymic nude mice bearing LNCaP prostate cancer tumors, administered either [^188^Re]Re-DP1-PSMAt or [^188^Re]Re-DP2-PSMAt at 2 h after injection (A), at 24 h after injection (B), and at 2 h after injection (C) with coadministration of Gelofusine. (D) Additionally, biodistributions of [^99m^Tc]Tc-DP1-PSMAt and [^99m^Tc]Tc-DP2-PSMAt at 2 h after injection were measured (Supplemental Tables 5–8). GI = gastrointestinal tract.

With a view to developing a molecular [^188^Re]Re-labeled agent for PSMA PRRT, we further characterized the biodistribution profiles of [^188^Re]Re-DP1-PSMAt and [^188^Re]Re-DP2-PSMAt in male athymic nude mice bearing LNCaP xenografts (Fig. 4), which recapitulate clinical metastatic prostate cancer more closely than DU145-PSMA+ xenografts. Importantly, the ^188^Re agents exhibited significant retention in tumors up to at least 1 d after administration. In mice administered [^188^Re]Re-DP1-PSMAt, LNCaP tumor uptake measured 7.0 ± 2.3 %ID/g at 2 h after injection and 2.9 ± 0.8 %ID/g at 24 h after injection. For [^188^Re]Re-DP2-PSMAt, LNCaP tumor uptake measured 9.8 ± 2.8 %ID/g at 2 h after injection and 7.6 ± 4.4 %ID/g at 24 h after injection. With the exception of the kidneys, radioactivity concentrations in other organs were similar to what was observed in prior experiments with male SCID/beige mice.

The concentration of ^188^Re agents in the kidneys was relatively high at 2 h after injection: [^188^Re]Re-DP1-PSMAt measured 137.1 ±34.6 %ID/g, and [^188^Re]Re-DP2-PSMAt measured 162.8 ± 31.8 %ID/g. These decreased to 29.4 ± 4.9 %ID/g and 37.0 ± 12.2 %ID/g, respectively, at 24 h after injection. Separate groups of animals were coadministered the plasma expander Gelofusine, which has previously been used to reduce kidney retention of PRRT agents and minimize potential nephrotoxicity. For animals coadministered [^188^Re]Re-DP1-PSMAt and Gelofusine, kidney retention decreased to 108.8 ± 13.3 %ID/g at 2 h after injection (*P* = 0.11); for [^188^Re]Re-DP2-PSMAt, kidney retention decreased to 127.6 ± 17.7 %ID/g (*P* = 0.046).

Last, to further assess the biologic equivalence of pairs of ^99m^Tc and ^188^Re agents, the biodistributions of [^99m^Tc]Tc-DP1-PSMAt and [^99m^Tc]Tc-DP2-PSMAt were also assessed in athymic nude mice bearing LNCaP prostate cancer tumors. Importantly, the biodistribution patterns and clearance pathways of [^99m^Tc]Tc-DP1-PSMAt and [^188^Re]Re-DP1-PSMAt were highly similar when compared in the same mouse model (either male athymic nude mice bearing LNCaP xenografts or male SCID/beige mice bearing DU145-PSMA+ xenograft tumors). The same near-equivalent biodistribution patterns were observed for [^99m^Tc]Tc-DP2-PSMAt and [^188^Re]Re-DP2-PSMAt. Between analogous ^99m^Tc and ^188^Re radiotracers, the most notable difference in biodistribution behavior was that ^99m^Tc radiotracers demonstrated consistently higher kidney residualization than did ^188^Re radiotracers.

## DISCUSSION

Theranostic PSMA-targeted radiopharmaceuticals have had an extraordinary impact on prostate cancer care in health care settings where they are available. We have developed 2 pairs of chemically analogous theranostic agents based on the generator-produced radionuclides ^99m^Tc and ^188^Re. Radio-HPLC alongside careful chemical characterization of nonradioactive or long-lived isotopologs (17) demonstrates that technetium and rhenium pairs are chemical analogs and isostructural. Consequently, each pair exhibits highly similar biologic behavior in in vitro and in vivo models of prostate cancer. Importantly, both ^188^Re- and ^99m^Tc-labeled complexes of DP1-PSMAt and DP2-PSMAt show a high accumulation in PSMA-expressing tumors and prostate cancer cells and, in vivo, rapidly clear from the circulation via a renal pathway, with minimal retention in healthy tissues. Furthermore, in vivo, these 4 radiotracers all demonstrate significant retention in prostate cancer tumors up to 24 h after injection.

In a clinical context, the favorable and near-identical biologic behaviors of chemically analogous ^99m^Tc and ^188^Re radiotracers bring about the possibility of using ^99m^Tc molecular imaging to predict the biodistribution, accumulation, and dosimetry of a complementary ^188^Re PRRT agent. Our novel theranostic pairs, M-DP1-PSMAt and M-DP2-PSMAt (M = [^99m^Tc]Tc, [^188^Re]Re), which use economical generator-produced isotopes, have strong potential utility for this purpose in prostate cancer treatment, particularly in LMICs. Additionally, the DP chemical platform underpinning these theranostic radiotracers is an excellent candidate for development of other peptide-based radiotracers: this technology could increase clinical use of receptor-targeted ^99m^Tc and ^188^Re radiopharmaceuticals and widen patient access to the benefits of molecular theranostic agents.

In this work, [^99m^Tc]Tc-DP1-PSMAt, [^99m^Tc]Tc-DP2-PSMAt, [^188^Re]Re-DP1-PSMAt, and [^188^Re]Re-DP2-PSMAt are all subject to postsynthetic HPLC purification and isolation procedures. Using HPLC, all these compounds can be easily separated from unreacted ligand and from unreacted ^99m^Tc/^188^Re-labeled precursors, yielding radiotracers of extremely high specific activity; that is, with the exception of decay products, there is minimal unlabeled DP1-PSMAt or DP2-PSMAt present in final radiotracer formulations. This culminates in an extremely high accumulation in PSMA-expressing prostate cancer cells in vitro and tissue in vivo (tumors, spleen, and in the case of DP1-PSMAt derivatives, kidneys).

In some in vivo experiments, there were statistically significant differences in tumor uptake between DP1 and DP2 bioconjugate derivatives; however, these differences were not recapitulated in alternative experiments. For example, in experiments using SCID/beige mice bearing DU145-PSMA+ tumors at 2 h after injection, tumors had significantly higher [^99m^Tc]Tc-DP2-PSMAt (29.4 ± 6.3 %ID/g) than [^99m^Tc]Tc-DP1-PSMAt (18.0 ± 3.5 %ID/g; mean difference, 11.4 %ID/g; *P* = 0.01). However, in experiments using athymic nude mice bearing LNCaP tumors, no statistically significant difference in tumor uptake was observed between these 2 ^99m^Tc radiotracers at 2 h after injection. Between homologous DP1 and DP2 radiotracers, the most notable difference was that M-DP1-PSMAt (M = [^99m^Tc]Tc or [^188^Re]Re) exhibited significantly higher spleen retention than did M-DP2-PSMAt at 2 h after injection (for comparisons of [^99m^Tc]Tc DP1-PSMAt vs. [^99m^Tc]Tc DP2-PSMAt or of [^188^Re]Re-DP1-PSMAt vs. [^188^Re]Re-DP2-PSMAt in SCID/beige or athymic nude mice, *P* ≤ 0.05).

All 4 radiotracers exhibited high kidney residualization. In the case of DP1-PSMAt derivatives, it is likely that this retention is in part mediated by PSMA (in SCID/beige mice, [^99m^Tc]Tc-DP1-PSMAt: 116 ± 27 %ID/g, with PMPA: 38 ± 2.9 %ID/g, *P* = 2.88 × 10^−3^; [^188^Re]Re-DP1-PSMAt: 88 ± 19 %ID/g, with PMPA: 38 ± 3.4 %ID/g, *P* = 1.16 × 10^−2^). Murine kidney tissues, specifically proximal renal tubules and Bowman capsule, express PSMA (23). DP2 radiotracers consistently demonstrated higher kidney retention than DP1 radiotracers. We attribute this to the comparatively higher lipophilicity of DP2 derivatives (17). Prior comparative in vivo murine studies have observed that increases in the lipophilicity of PSMAt-derived compounds increase their kidney retention (24). Although coadministration of Gelofusine decreased kidney retention of ^188^Re agents at 2 h after administration, this observed decrease was not statistically significant. In light of the comparatively similar tumor uptake of DP1 and DP2 radiotracers but the higher kidney retention of DP2 radiotracers, we postulate that DP1-based radiotracers are better clinical theranostic candidates.

Compared with the existing ^99m^Tc- and ^188^Re-radiolabeled tracers—[^99m^Tc]Tc-MIP-1404 (5), [^99m^Tc]Tc-PSMA-I&S (6), [^99m^Tc]Tc-EDDA/HYNIC-iPSMA (9), and [^188^Re]Re-PSMA-GCK01 (16)—the new DP-based radiotracers demonstrate either decreased or comparable residualization in murine liver and either increased or comparable blood clearance at 1–2 h after administration (Supplemental Table 2).

Like existing PSMA-targeted ^99m^Tc radiotracers, both [^99m^Tc]Tc-DP1-PSMAt and [^99m^Tc]Tc-DP2-PSMAt can be formulated using a kit: our existing protocols and prototype kit radiosynthetic methods enable high radiochemical yields of desired radiotracer (80%–90%) by heating a saline solution of generator-produced [^99m^Tc]TcO_4_^−^ with kit components at 100°C for 5 min (17–19). We elected to use a 2-step protocol to prepare ^188^Re-radiolabeled agents, in which a [^188^Re]Re^V^-citrate precursor is reacted with either DP1-PSMAt or DP2-PSMAt to yield the desired ^188^Re radiotracers. This procedure is similar to prior radiosyntheses of [^188^Re][Re^V^O_2_]^+^-labeled phosphine-containing P_2_S_2_ and P_2_N_2_ tetradentate chelator derivatives (20,21). However, complexes of the latter chelators were obtained in more than 90% radiochemical yield; the highest radiochemical yields obtained for [^188^Re]Re-DP1-PSMAt and [^188^Re]Re-DP2-PSMAt were about 70% and 50%, respectively. We also note that the [^188^Re][ReO^V^]^3+^-based complex, [^188^Re]Re-PSMA-GCK01, can be obtained in 78% radiochemical yield and 96% radiochemical purity, using a one-pot radiochemical procedure (16). This procedure involves heating an aqueous solution of precursor GCK01, [^188^Re]ReO_4_^−^, citrate, and ascorbic acid at pH 2.0–3.5 at high temperature for 1 h, followed by neutralization, further heating for 5 min, and final C18 Sep-Pak purification. We are currently optimizing kit-based formulations to increase the radiochemical yields of DP-based ^99m^Tc and ^188^Re radiotracers and to obviate postsynthetic purification procedures to remove unreacted ^99m^Tc and ^188^Re precursors.

## CONCLUSION

We have developed new PSMA-targeting ^99m^Tc/^188^Re theranostic agents, using versatile diphosphine chemical platforms. These radiotracers can be prepared using eluate from bench-top ^99m^Tc and ^188^Re generators and chemical kits. The resulting isostructural ^99m^Tc and ^188^Re pairs show near-equivalent biologic behaviors in models of prostate cancer. We are further developing optimized kit-based formulations to enable near-quantitative radiochemical yields to obviate purification steps after radiolabeling. Our new, generator-based theranostic agents have potential to provide access to the benefits of PSMA-targeted diagnostic imaging and systemic radiotherapy in health care settings that do not routinely have access to either reactor-produced ^177^Lu radiopharmaceuticals or PET/CT infrastructure.

## DISCLOSURE

This research was supported by a Cancer Research UK. Career Establishment Award (C63178/A24959), an EPSRC program grant (EP/S032789/1), the Cancer Research UK. National Cancer Imaging Translational Accelerator Award (C4278/A27066), and the Wellcome Trust (212885/Z/18/Z). The authors have submitted a patent application describing the intellectual property described in this article. No other potential conflict of interest relevant to this article was reported.

## Notes

KEY POINTS **QUESTION:** Are molecular theranostic radiotracers based on ^99m^Tc and ^188^Re feasible for diagnostic imaging and systemic radiotherapy of prostate cancer? **PERTINENT FINDINGS:** Pairs of chemically analogous ^99m^Tc/^188^Re radiotracers show equivalent uptake in PSMA-expressing prostate cancer cells and favorable, highly similar biodistribution profiles in mouse models of prostate cancer. **IMPLICATIONS FOR PATIENT CARE:** These generator-based theranostic agents have potential to provide access to the benefits of PSMA-targeted diagnostic imaging and systemic radiotherapy in health care settings that do not routinely have access to either cyclotron- or reactor-produced radionuclides.
